# Immediate and Long-Term Outcome of Acute H_2_S Intoxication Induced Coma in Unanesthetized Rats: Effects of Methylene Blue

**DOI:** 10.1371/journal.pone.0131340

**Published:** 2015-06-26

**Authors:** Takashi Sonobe, Bruno Chenuel, Timothy K. Cooper, Philippe Haouzi

**Affiliations:** 1 Department of Medicine, Division of Pulmonary and Critical Care Medicine, Pennsylvania State University, College of Medicine, Hershey, PA, United States of America; 2 Department of Comparative Medicine, Pennsylvania State University, College of Medicine, Hershey, PA, United States of America; 3 Department of Pathology, Pennsylvania State University, College of Medicine, Hershey, PA, United States of America; Emory University, UNITED STATES

## Abstract

**Background:**

Acute hydrogen sulfide (H_2_S) poisoning produces a coma, the outcome of which ranges from full recovery to severe neurological deficits. The aim of our study was to 1- describe the immediate and long-term neurological effects following H_2_S-induced coma in un-anesthetized rats, and 2- determine the potential benefit of methylene blue (MB), a compound we previously found to counteract acute sulfide cardiac toxicity.

**Methods:**

NaHS was administered IP in un-sedated rats to produce a coma (n = 34). One minute into coma, the rats received MB (4 mg/kg IV) or saline. The surviving rats were followed clinically and assigned to Morris water maze (MWM) and open field testing then sacrificed at day 7.

**Results:**

Sixty percent of the non-treated comatose rats died by pulseless electrical activity. Nine percent recovered with neurological deficits requiring euthanasia, their brain examination revealed major neuronal necrosis of the superficial and middle layers of the cerebral cortex and the posterior thalamus, with variable necrosis of the caudate putamen, but no lesions of the hippocampus or the cerebellum, in contrast to the typical distribution of post-ischemic lesions. The remaining animals displayed, on average, a significantly less effective search strategy than the control rats (n = 21) during MWM testing. Meanwhile, 75% of rats that received MB survived and could perform the MWM test (P<0.05 vs non-treated animals). The treated animals displayed a significantly higher occurrence of spatial search than the non-treated animals. However, a similar proportion of cortical necrosis was observed in both groups, with a milder clinical presentation following MB.

**Conclusion:**

In conclusion, in rats surviving H_2_S induced coma, spatial search patterns were used less frequently than in control animals. A small percentage of rats presented necrotic neuronal lesions, which distribution differed from post-ischemic lesions. MB dramatically improved the immediate survival and spatial search strategy in the surviving rats.

## Introduction

One of the most impressive clinical features of hydrogen sulfide (H_2_S) intoxication in humans is certainly a phenomenon referred to as “knockdown” [1–3]. The term has been coined to describe a clinical picture, which typically consists in a sudden loss of consciousness in a subject exposed to toxic levels of H_2_S. This coma can be associated with a cardiorespiratory depression, which in the most severe forms can be lethal [4, 5] or lead to severe neurological sequelae [6–8]. However, if the subject is withdrawn from the source of exposure, or the exposure of H_2_S ceases before a cardiac shock develops, the coma can be rapidly and spontaneously reversible and very few after-effects are thought to develop if consciousness is regained rapidly [9]. These observations have raised the question of whether and at which level H_2_S, by itself, could produce some direct neuronal toxicity, i.e. without the presence of a cardiorespiratory depression. This question has already been partly addressed by Baldelli et al. [10] in a study wherein H_2_S induced coma was produced in un-anesthetized versus mechanically ventilated rats. In non-lethal forms of sulfide poisoning, H_2_S induced neuronal lesions seems to be potentiated by the presence of a concomitant cardiorespiratory failure [10]. Similarly, the severe neurological sequelae described in patients following H_2_S-induced coma concern individuals requiring cardiorespiratory support and who presented with severe shock, acute respiratory failure and prolonged coma [7, 8].

Various agents have been proposed to treat H_2_S poisoning [1, 5, 11–15], most of them with the theoretical purpose of trapping and/or “oxidizing” free H_2_S with metallo-compounds, e.g. ferric iron produced by nitrite-induced methemoglobinemia [16–20] or cobalt in Hydroxocobalamin (vitamin B12) [21, 22]. Other antidotes are based on empirical observations, such as sodium bicarbonate [23], hyperoxia [24, 25], or methods using reducing agents to “remove” sulfide from cysteine residues [26]. There is still no consensus on the effective treatment to be used. Indeed, the main limit of using specific antidotes against H_2_S poisoning aimed at trapping sulfide is that soluble/diffusible H_2_S disappears very rapidly (in sometimes less than one minute) and spontaneously *following* the cessation of H_2_S exposure [27]. Their efficacy is therefore very limited as sulfide susceptible to be trapped after an exposure vanishes so quickly. New paradigms must be proposed using agents correcting the consequences of H_2_S toxicity, rather than trying to trap soluble H_2_S [28]. As developed in the discussion section, we have previously shown that methylene blue (MB) or Azure B, two phenothiazinium chromophores [29], counteract the rapid depression in cardiac contractility produced during and following acute sulfide intoxication [28]. The possible beneficial mechanisms of MB during H_2_S includes the support of mitochondrial respiration [30–33], as well as potent antioxidant [30, 33–36] and anti-NO properties [37, 38], which could counteract the effects of H_2_S [39]. The effects of MB could also be beneficial for the neurological outcome, akin to the remarkable protection of MB against the toxic effects of Sodium Azide (SA) [40], which, like H_2_S, is a poison of the mitochondrial activity. This beneficial effect has been observed when MB was injected only once after SA exposure [40], or for a few days during chronic SA exposure [41].

Un-anesthetized models of H_2_S induced coma have already been used in the rat [10, 42]. They certainly represent, in contrast to mice [43, 44], a clinically relevant approach faithful to human sulfide poisoning. In the present study, we have intended to characterize the natural history and neurological outcome of H_2_S induced coma using a modified version of a model we have previously developed in un-anesthetized rats [28]. Our aims were to 1) characterize the range of manifestations and outcomes of H_2_S induced coma in keeping with the effects reported in humans, i.e. rapid recovery, survival with profound deficits or rapid death 2) describe the cognitive and memory functions as well as the anatomical distribution, the frequency of occurrence and the specificity of neuronal lesions, if any, in rats spontaneously surviving the initial period of coma; 3) evaluate the effects of methylene blue (MB).

## Material and Methods

### Animals

Male Sprague-Dawley rats (Charles River) weighing 471 ± 77 g (~12–16 weeks) were studied. All experiments were conducted in accordance with the Guide for the Care and Use of Laboratory Animals, 8^th^ Edition (National Research Council (US) Institute for Laboratory Animal Research). The study was approved by the Pennsylvania State University College of Medicine Institutional Animal Care and Use Committee (reference number 44716). All rats were housed in open top cages in a temperature-controlled conventional room (24 ± 1°C) with 12-hour light/dark cycle, and were provided with standard laboratory diet and water ad libitum.

### Protocol of NaHS-induced coma

We have previously described a protocol of NaHS induced coma [28]. This initial protocol was modified as followed: sulfide was administered intraperitoneally, as a solution of sodium hydrosulfide hydrate (NaHS, Sigma Aldrich, St Louis, MO) prepared, in sterile physiological saline (5 mg/ml), immediately prior to each experiment and kept in airtight syringes. We found that IP administration of 20 mg/kg NaHS led to a rapid coma in about one third of the rats after one injection. Interestingly, a second injection performed 10 minutes later, produced a coma in two thirds of the remaining rats. Concentrations higher than 20 mg/kg at once produced coma leading to death in most cases, while lower doses produce brief episodes of somnolence but rarely a coma. The following protocol was therefore chosen: A first injection of NaHS was administered in 34 rats total; the animals that did not present a coma within 10 minutes (coma occurs within 3 minutes) received a second dose. The same protocol was repeated one more time in refractory rats, in contrast to our initial description [28].

Clinical examinations were performed every minute for 30 minutes following NaHS administration. This examination included the determination of presence of the righting reflex, the response to hand clapping and air puff, the grasping reflex, and the withdrawal reflex [45]. Breathing pattern and the presence of a cardiac pulse were monitored. Coma was defined by the disappearance of response to clapping and air puff along with the loss of the righting reflex. Animals that displayed a phase of somnolence, while all the reflexes were still preserved were therefore not considered to present a coma. Corneal reflex was tested as soon as the animal was in coma. Breathing movements were monitored to identify tachypnea (rapid shallow breathing with a breathing frequency above 400/min), hypopnea (breathing frequency below 100/min) and production of abrupt and large breaths at a low frequency (gasping).

During the first 24 hours following NaHS injection, each animal was observed carefully for signs of distress or discomfort. Animals showing signs of prostration, inability to walk, to eat, drink, paralysis or visual deficit were euthanized at D2 and their brains and lungs were examined. The rats that survived the phase of coma and did not present any sign of deficit or discomfort within the following 24 hours were included in the second part of the study. Animals were weighed every day. Finally, a group of 21 rats matched to NaHS-injected rats received a saline IP injection instead of NaHS and were used as control.

### Methylene blue administration

Three hours before the experimental protocol, rats were briefly anesthetized using 1–2% isoflurane in 100% O_2_. A 22G IV catheter (Jelco, Smiths Medical) was placed into one of the lateral tail veins. An injection line (Medline) was inserted into the exteriorized end of the cannula and heparinized. The catheter was protected by a plastic tube placed around the tail. The catheter was removed at the end of protocol.

According to the protocol described above, the treated animals (see below for n) received 1 ml of MB solution (4 mg/kg MB, Akorn, Inc., Lake Forest, IL), as previously described [28], as soon as large and slow breaths were produced (See result section), typically 60 seconds into the phase of coma. A 2^nd^ injection of MB was always performed 1 minute later.

### Morris water maze task

**Fig 1:**
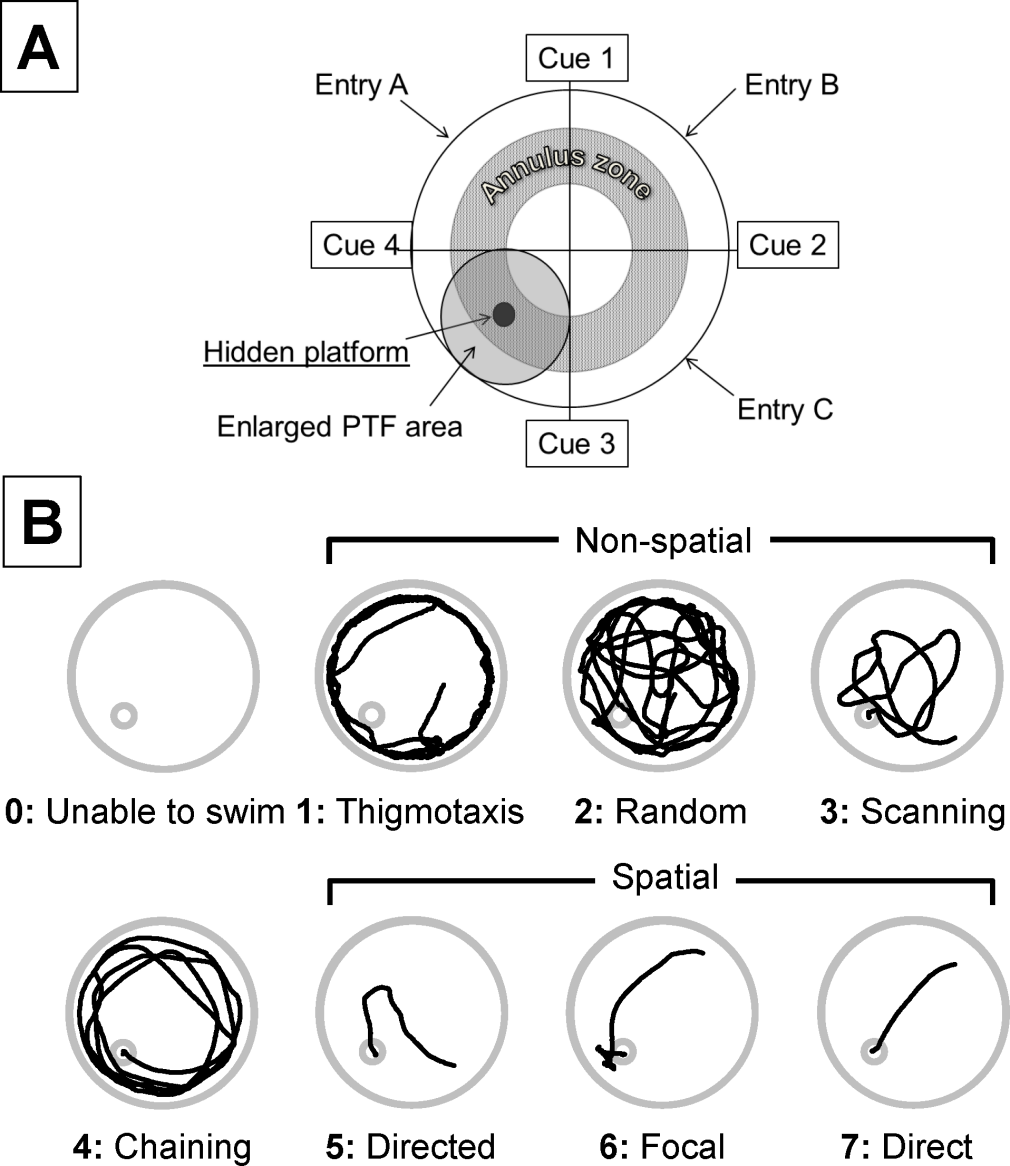
Classification of swimming strategies. A) Schematic drawing of the water maze pool setting showing the location of platform and cues. Animals were placed at either of 3 entries of the pool. The annulus zone and the large circular area around the platform (PTF) were used to identify thigmotaxis, chaining or focal search as previously descried [54]. B) Swimming strategies used by the rats were classified in 8 categories (based on modified criteria proposed by Garthe et al. [54]) and were given a score from 0 to 7. Thigmotaxis, Random search and Scanning are regarded as spatial memory independent, or non-spatial strategies, while Directed, Focal, and Direct search were considered as spatial memory dependent. Chaining was scored separately. A score of 0 was given to the animals unable to swim. The traces are actual swimming patterns observed in our control rats.

Twenty four hours following the episode of NaHS-induced coma, the surviving rats, as well as the control/saline rats, were trained in the reference memory version of the Morris water maze (MWM) task [46–48] to locate a hidden transparent platform (11 cm diameter, 1.5 cm below surface) in a circular pool (1.80 m diameter, 60 cm height). The room was equipped with four extra-maze cues (60 × 70 cm) located on the four directions (S, N, E, W) behind the maze’s wall, to facilitate spatial learning. Water was kept at a temperature of 19–20°C. Each rat was given 4 trials per day for 4 consecutive days with an inter-trial interval of 10 min. Platform position remained constant throughout the study and was located in the SW quadrant (Fig 1). Rats were released from one of three other quadrants and allowed to search the platform up to 120 s. Every day, the starting position was changed in a random manner. On the first day, rats that did not find the platform were guided to the platform and were allowed to remain there for at least 30 sec. Swim paths of each animal on each trial were recorded using a ceiling-mounted digital camera and ANY-maze video tracking software (version 4.99, ANY-maze, Stoelting Co., Wood Dale, IL) for further analysis. On the last day, the fourth trial was replaced by a 120 s probe trial, i.e. with the platform removed. The probe trial provides a measure of retention, which is determined by time and distance swum to reach the site where the platform had been located and the amount of time spent in the enlarged platform area [49].

### Open field study

Spontaneous motor activity was also determined in the 3 groups of rats. Each animal was placed into a clear 100 cm × 100 cm polycarbonate cage with two photo-beams (ANY-maze Interface Photo-beam array, 100 cm, Stoelting and Co, Wood Dale, IL). The spontaneous locomotor activity and the number of times the animal would stand on his hind limbs were determined [50] for 10 min using an automated open field activity, with ANY-box system (Stoelting and Co, Wood Dale, IL). Animals were recorded using a ceiling-mounted digital camera and ANY-maze video tracking software (version 4.99, ANY-maze, Stoelting Co., Wood Dale, IL).

### Euthanasia and Brain Studies

One week after NaHS injection, the surviving rats were anesthetized with 3.5% isoflurane in O_2_ followed by urethane (1.2 g/kg, IP). Thirty minutes later, the animals were perfused trans-cardially with phosphate buffered saline followed by 10% neutral buffered formalin (NBF). In addition, as mentioned earlier, any moribund rats presenting a motor or sensory deficit within the first 24 hours of NaHS exposure were sacrificed at D2 using the same protocol. The head of the animal was skinned, decapitated and immersion fixed for 24 hours in 10% NBF before brain removal. The brains were fixed for an additional five days before trimming into nine 5-mm coronal slices [51, 52] using a brain-trimming matrix (Zivic). Tissues were processed in an automated Tissue-Tek VIP processor and paraffin-embedded with a Tissue-Tek TEC embedding station (Sakura Finetek USA, Torrance, CA). Sections were cut at 6 μm for routine hematoxylin and eosin (H&E) staining or were mounted on charged plus slides for immunohistochemistry (IHC). The brains of 2 of the saline rats were used as control.

All tissues were examined by an ACVP pathologist blinded to treatment. All images were obtained with an Olympus BX51 microscope and DP71 digital camera using cellSens Standard 1.6 imaging software (Olympus America, Center Valley, PA).

For immunohistochemistry, slides were deparaffinized and heat-induced antigen retrieval was performed in citrate buffer. Endogenous peroxide was blocked, and slides were incubated for 1 hour at room temperature with rabbit polyclonal antibodies to glial fibrillary acidic protein (GFAP, Abcam ab7260, 1:4000) or cleaved caspase-3 (CC3, Cell Signaling 9661, 1:500) or goat polyclonal to ionized calcium binding adaptor molecule-1 (Iba1, Abcam ab5706, 1:500), followed by biotinylated secondary antibody using a Vector Elite ABC kit and DAB chromagen. Slides were counterstained with Mayer’s hematoxylin. This approach was used to determine in 4 rats exposed to sulfide with no obvious neuronal necrosis 1- possible changes in populations of astrocytes and microglia and 2- the presence of markers of apoptosis.

### Data analysis

The primary outcomes of the coma (Control, H_2_S and H_2_S-MB) were 1- the immediate mortality, 2- the presence of obvious clinical abnormal behavior within 24 and 48 hours, 3- in the surviving animals: weight loss, difference in behavior in the open field activity and in the Morris Water Maze and 4- presence and location of neuronal necrosis.

For the Morris water maze analysis, the following parameters were analyzed: latency to reach the hidden platform, swim path length, and path efficiency (ratio of the actual swim path length to the ideal path that the animal could take to reach the submerged platform). In addition, search strategies to locate the hidden platform were analyzed following the description in mice [53–55]. Eight different strategies were described in our rat population as shown in Fig 1. Briefly, swim path data from ANY-maze video tracking (Stoelting and Co, Wood Dale, IL) were used to derive the time-tagged x-y coordinates. The respective predominant search strategy was then determined based on visual criteria by two different blinded observers (Fig 1). A third observer was systematically involved in the classification process whenever the two observers did not agree, and a consensus between the 3 observers was found. The spectrum of different search patterns spanned from initially undirected (from thigmotaxis to chaining) to spatially precise, highly efficient and allocentric strategies (form directed search to direct swimming) as illustrated in Fig 1. For convenience we have defined direct swimming, focal search and directed search as spatial categories, while non-spatial strategies comprised scanning, random search and thigmotaxis. Since chaining strategy could be considered as a transition mode between non-spatial and spatial or as a mode used by some over trained animals, this pattern was considered as a specific category. Criteria used to delineate the different pattern followed those previously established [53–55].

For spontaneous activity in the open field, the total distance and number of photo-beam breaks (upright position) were regarded as a marker of general activity.

### Statistical Analysis

All results are presented as mean ± SD. The daily changes in all variables of interest (body weight, latency to the platform, path length, path efficiency, patterns of search during water maze test) within a group over time were analyzed using one-way repeated-measured ANOVA followed by Bonferroni’s post-hoc comparisons. All variables of interest were also compared between the saline group or MB treated vs non-treated animals, using a two-way repeated ANOVA followed by Bonferroni’s post-hoc comparisons. The occurrence of deaths and each strategy used (spatial vs non-spatial) during the MWM testing were compared using a Chi square test. P<0.05 was regarded as significant for any of these comparisons. All statistical analyses were conducted using GraphPad Prism 6 (Graphpad Software, La Jolla, CA).

## Results

**Fig 2:**
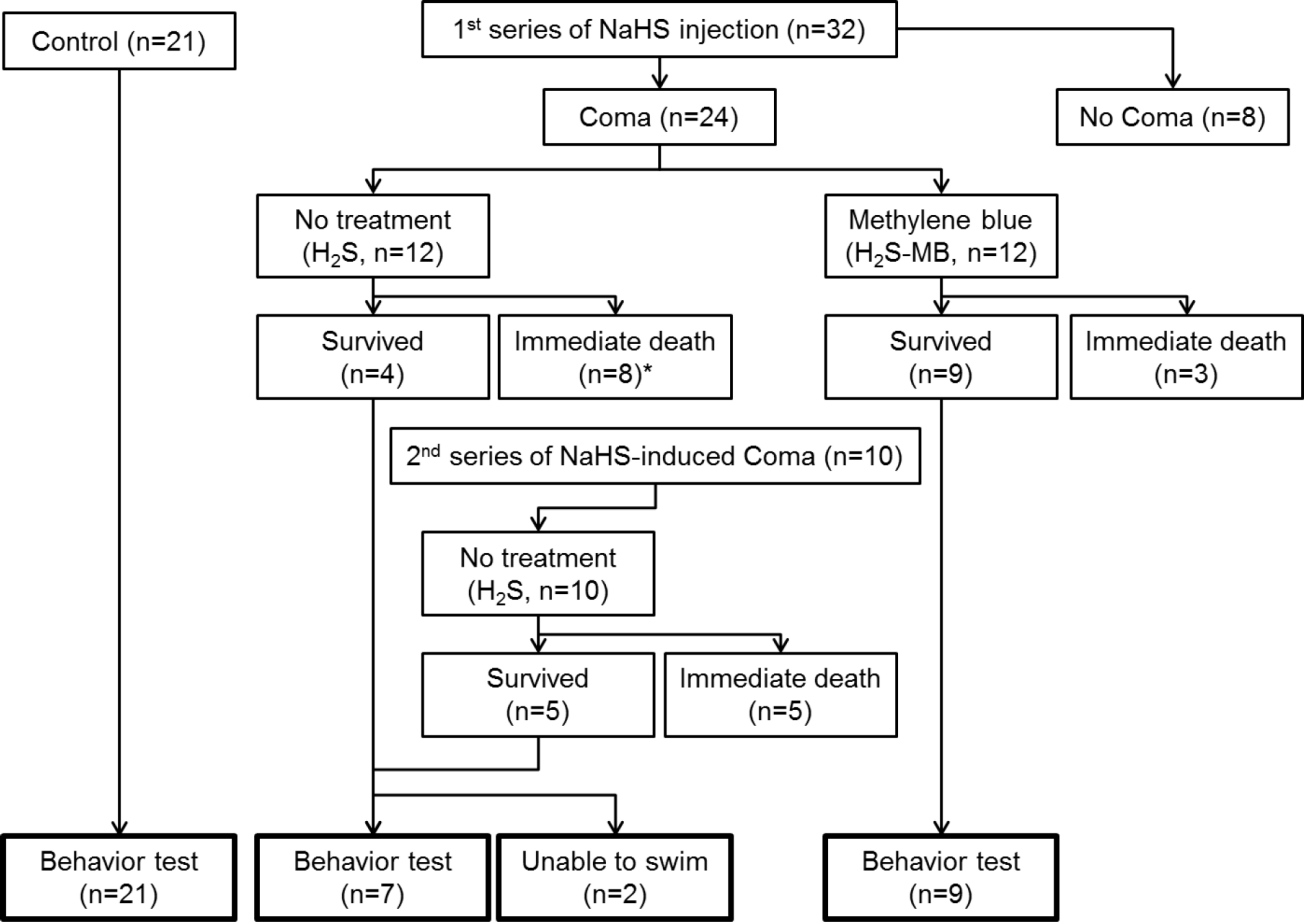
Experimental design and outcomes. Twenty-one control rats received saline IP injection and were assigned to behavior test (control rats). A first series of 32 rats received H_2_S injection, 8 rats did not present a coma. The 24 rats that presented a coma, were separated into a non-treatment (H_2_S) group (12 rats) and methylene blue treated (H_2_S-MB) group (12 rats). In the H_2_S group, 8 died immediately (*1 out of 8 rats died within 12 h) and 4 survived from a coma (one these 4 rats was however unable to swim). Meanwhile 9 survived in the H_2_S-MB group, they were all able to swim. Therefore, to match the number of surviving rats between the 2 groups, a second series of 10 rats received NaHS: 5 died and 5 survived. The 5 surviving rats were added to the H_2_S group, one of them could not swim. Out of 9 surviving rats in the H_2_S group, 7 could therefore complete the behavior test, while the 2 rats unable to swim were euthanized within 48 h. Out of 9 surviving rats in the H_2_S-MB group, 9 completed the behavior test.

The overall immediate outcomes are displayed in Fig 2.

### 1- NaHS-induced coma

#### a- Description of H_2_S induced coma

**Fig 3:**
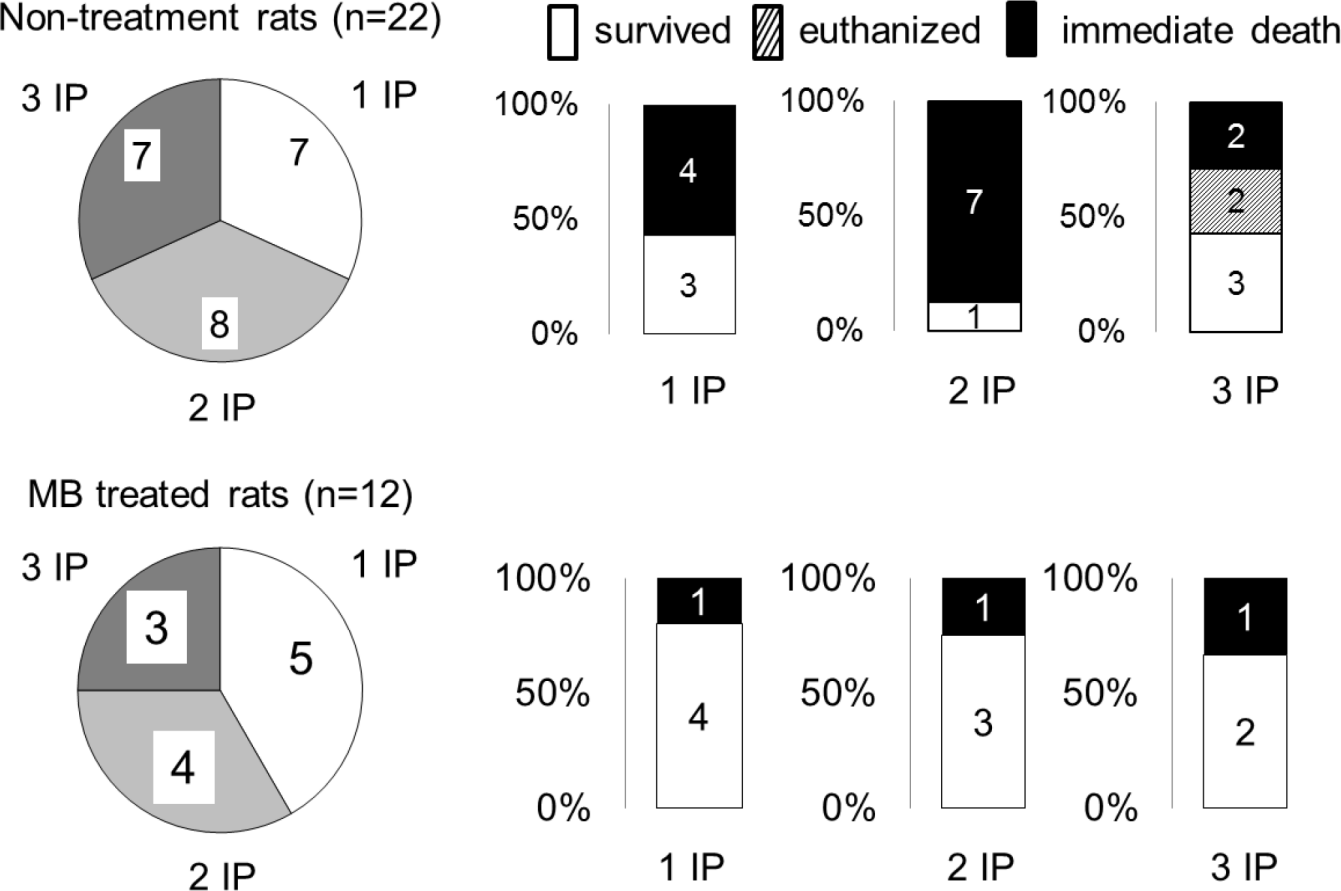
Immediate outcome in keeping with the number of intra-peritoneal NaHS injections required to produce a coma. There was no difference in the mortality whether 1, 2 or 3 injections were administered. Of note is that the 2 rats that were unable to swim in the H_2_S groups belonged to the rats that received 3 IP injections.

Whenever a coma occurred, its characteristics were very stereotypical and similar whether the coma was produced after one, two or three NaHS injections (Fig 3). Typically within one minute following NaHS administration, rats started to present signs of motor agitation, grooming their nose and eyes, along with a tachypnea, while a typical smell of rotten-eggs was detectable. Following this initial phase, all animals rapidly (i.e. within one to 2 minutes) stopped their spontaneous locomotor activity with a phase of muscle hypotonia, starting with the forelimbs. The rats lost their righting reflex and became unresponsive (loss of response to hand clapping or air puffing) around 3 min. Then the animals clearly reduced their breathing frequency leading to irregular ventilation. The latter led to recurrent episodes of apnea and gasping (large and slow breaths). This severe depression in breathing was very often associated with a loss of the corneal reflex. The mechanism of death was a cardiac arrest (disappearance of cardiac pulses) that occurred during this phase of gasping (around 7 minutes after injection). In most rats, gasping was still produced while cardiac pulsations disappear. In the surviving rats, the phase of coma was rather short, lasting on average 3.8 ± 1.0 min (from 2 to 5.6 min). The animals remained unable to use their limbs for a longer period: forelimb hypotonia/paralysis, preventing any efficient grasping or grabbing, lasted 14.6 ± 6.8 min. The animals progressively recovered the mobility of the hindlimbs first, followed by the forelimbs.

#### b- General outcome of NaHS induced coma

The 21 rats that received saline instead of NaHS presented no sign of discomfort or deficit at any stage of the study. A first series of thirty-two rats received NaHS according to the protocol described in the methods section; of these, 24 rats presented a coma (Fig 2). These 24 rats were then separated into 2 groups, non-treatment (n = 12, H_2_S) or methylene blue-treated group (n = 12, H_2_S-MB). Among the 12 rats of the H_2_S group, 4 recovered from the coma, while 7 died within the first 10 minutes and one died within the first 8 hours (67% mortality). Three out of 4 surviving rats behaved normally without any clinical signs, while the remaining rat presented obvious clinical deficit (see below). In contrast, among the 12 rats of the H_2_S-MB group presenting a coma, 9 recovered with no obvious deficit at 24 hours, while 3 died immediately (25% mortality). The survival with no deficit in the H_2_S-MB group was significantly higher than in the H_2_S group (P<0.05). To achieve a similar number of surviving animals in both groups, 10 additional rats received an IP injection of NaHS; they all presented a coma (Fig 2).

The number of injections of NaHS required to get a coma was variable among the 34 rats: 12 rats presented a coma after a single injection (35%), 12 out of the 22 remaining rats presented a coma after a second injection (55% of the remaining rats), while the 10 remaining rats presented a coma after a third injection (100% of the remaining rats) (Fig 3). Of note is that the animals who did not present a coma after the 1^st^ (22/34) or 2^nd^ (10/22) NaHS injection, displayed a very transient reduction in their spontaneous locomotion along with a detectable smell of rotten-egg breath that was sensed by the examiner. These rats were drowsy for 15–30 seconds, but all reflexes remained intact and no motor deficit was observed. The animals that did not show a coma returned to a normal behavior with no exception within less than 2 minutes. We did not find any significant differences in the duration of H_2_S-induced coma among the groups in keeping with the numbers of injection. This remains true whether all the animals are included: 3.8 ± 1.3 min (1 injection), 4.9 ± 2.6 min (2 injections), 2.7 ± 1.8 min (3 injections) or only those surviving the coma are considered: 4.7 ± 1.5 min (1 injection), 2.5 min (2 injections), 2.0 ± 1.0 min (3 injections).

#### c- Immediate outcome: H_2_S vs H_2_S-MB

As presented in section 2a, out of a total 22 rats intoxicated with H_2_S, nine survived, while 13 rats died (60% immediate mortality, Fig 2). Seven out of the 9 surviving rats displayed a complete recovery of the phase of coma, with no obvious behavioral or motor deficit, after about 20 minutes. The 2 remaining rats remained extremely drowsy with a low reactivity to external stimuli for hours following NaHS injection. They all had required 3 injections to produce a coma (Fig 3). In these 2 rats, motor or sensory deficits were visible (Fig 2), one rat was clearly blind while the other had difficulty moving his forelimbs. In contrast to the 7 other surviving rats, these rats were unable to eat, drink or swim, requiring euthanasia within 48 hours. None of the animals displayed symptoms of respiratory distress, and any visible change in breathing pattern.

There was no difference in the time to coma between the H_2_S (3.0 ± 1.5 min) and H_2_S-MB groups (2.9 ± 0.9 min). The 12 rats that received MB displayed a bluish coloration of eyes and skin. As already mentioned above, 9 out of 12 animals did recover from the coma. All the surviving rats showed no obvious signs of clinical deficit at 24 h. None of the surviving rats displayed signs of respiratory distress, including tachypnea, bradypnea or labored0020breathing.

#### d- Body weight

**Fig 4:**
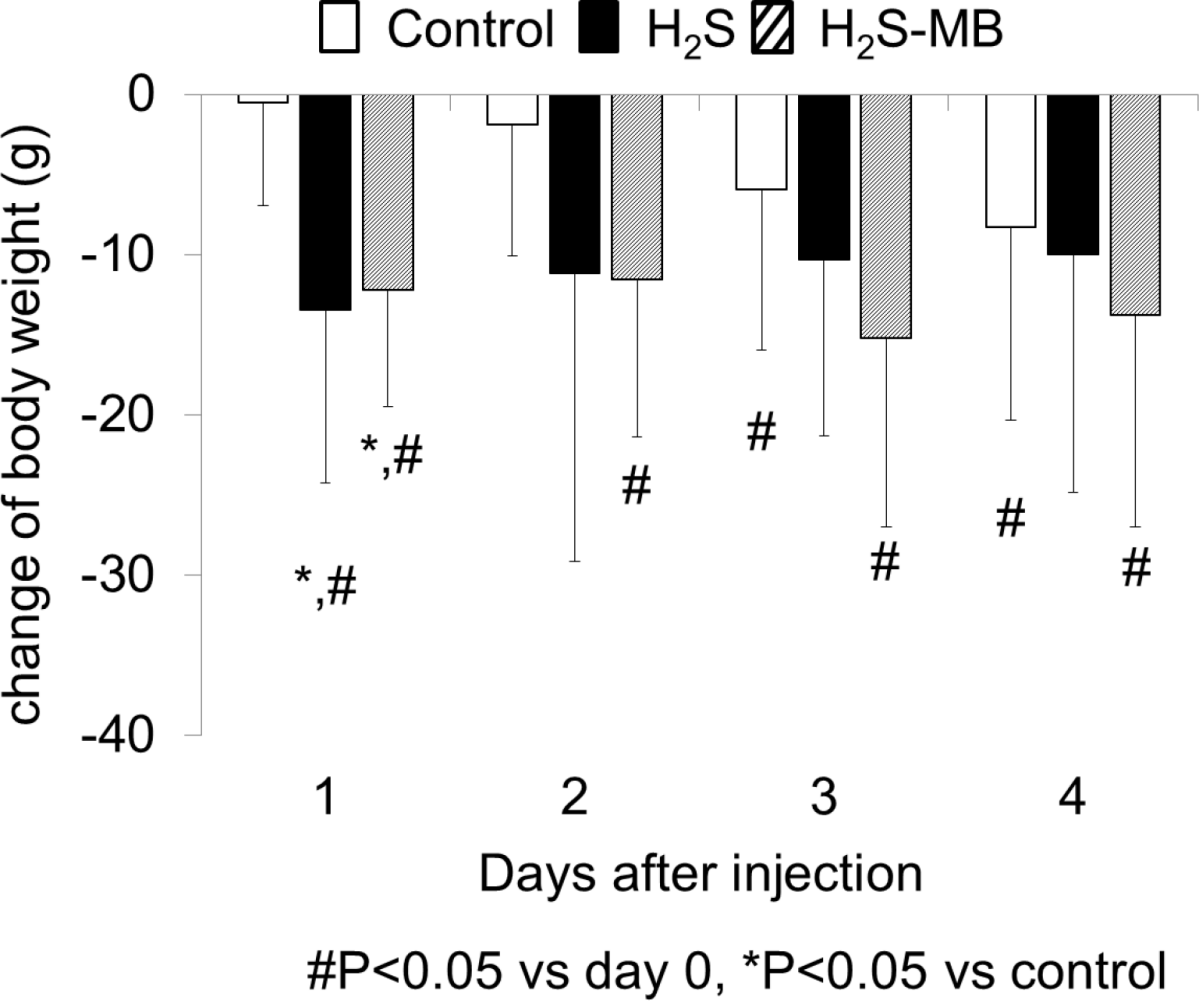
Body weight following H_2_S induced coma. Body weight decreased gradually over the 4 days of training in the control group. In the H_2_S group, body weight significantly dropped at D1 day (significantly different from control group, P<0.05), and then did not change thereafter. Note that the 2 rats unable to eat that were euthanized are not included in this computation. In the H_2_S-MB group, weight also significantly dropped at D1 (significantly different from control group, P<0.05) then remained below baseline until D4. Values are shown as mean ± SD. *significantly different from control at P<0.05. # Significantly different from baseline (day 0) at P<0.05.

The three groups of rats had a weight that was not significantly different at baseline. The weight of the surviving rats of the H_2_S group, that were able to swim, decreased from 471 ± 106 g to 457 ± 99 g at D1 (-2.6 ± 2.2% of the initial body weight, P<0.05 vs baseline and versus control group, Fig 4). The body weight of the 9 surviving rats in the H_2_S-MB group also decreased from 459 ± 52 g to 447 ± 51 g at D1 (-2.7 ± 1.6% of the initial body weight, P<0.05 vs baseline and vs control group, Fig 4). There was no further significant loss of weight after that. Of note is that the 21 rats that were used as control receiving saline injection (4 ml/kg, IP) displayed a light but significant progressive decrease in body weight from 484 ± 81 g to 476 ± 75 g at D4 of MWM testing (P<0.01, Fig 4).

#### e- Open field study

Spontaneous motor activity was determined for 10 minutes in 5 control rats. Typically, rats moved to a corner of the field when the test was initiated, then started walking along the side of wall or stopped for grooming. The averaged total traveling distance was 24.8 ± 10.0 m on D1 and 15.2 ± 13.8 m on D4 of the MWM study. The average number of photo-beam breaks was 20 ± 15 on D1 and 14 ± 12 on D4. There were no significant changes in the traveling distance and the number of photo-beam breaks in keeping with the day of study.

In the surviving rats of H_2_S group (not including the 2 rats with neurological deficit), the averaged total traveling distance was 10.2 ± 7.1 m, and the average number of photo-beam brake was 11 ± 8 for 10 min of observation at D4.

In the H_2_S-MB group, all of 9 rats showed a relatively more active behavior at D4, with a longer average traveling distance (32.1 ± 9.5 m, P<0.05 vs control and H_2_S groups) and a higher average number of photo-beam breaks (31 ± 21, P<0.05 vs H_2_S group).

#### f- Morris Water Maze

##### Quantitative evaluation

**Fig 5:**
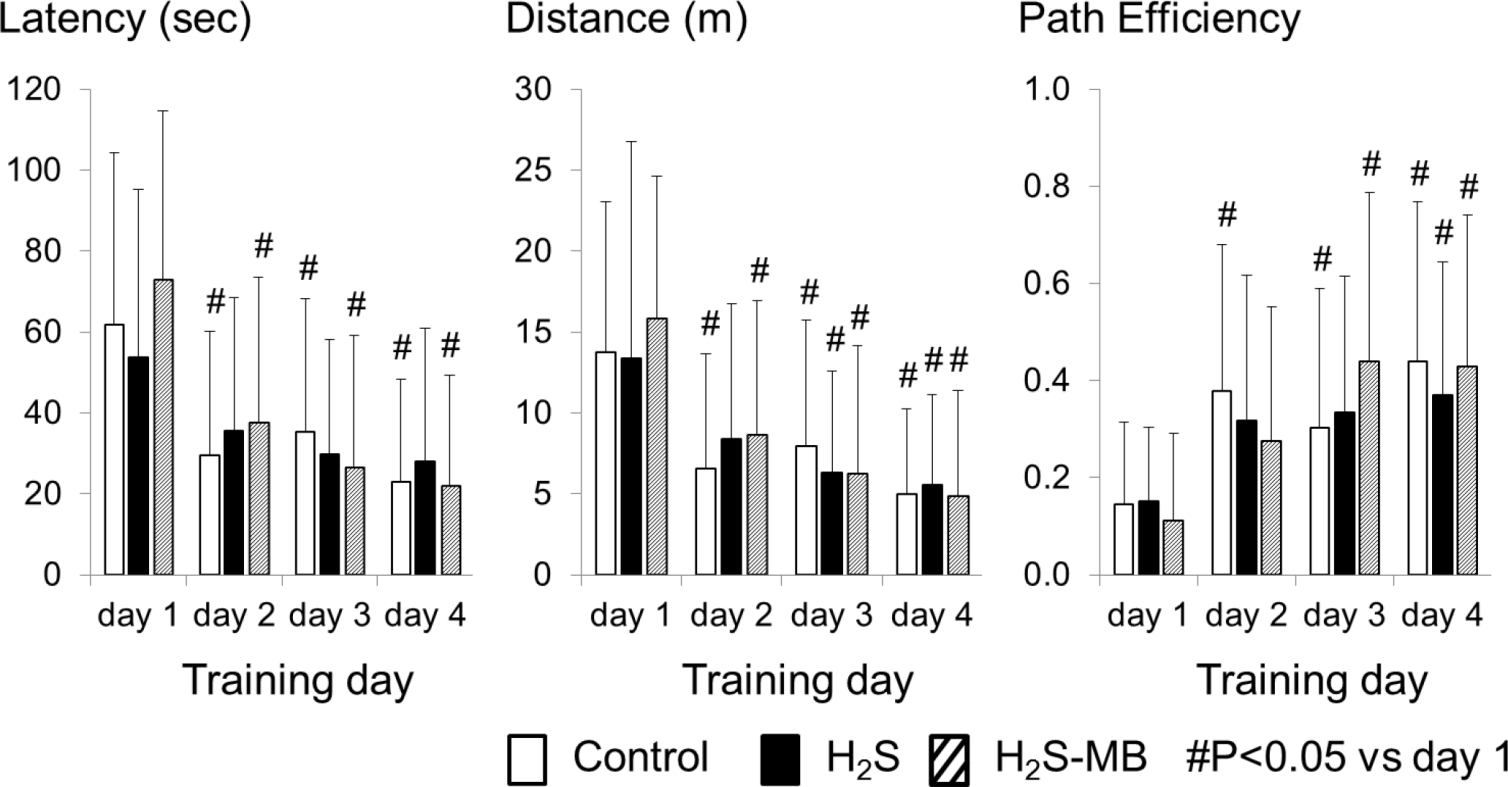
Latency to reach the platform, distance, and path efficiency during Morris water maze testing. Data shown here correspond to the results obtained in all the rats that were able to swim and to find the platform the 4 tests. All rats decreased the latency and distance to locate the platform and increased the path efficiency throughout the 4 days of training with no difference between groups. In the H_2_S group, the changes were significant only in for the distance at D3 and D4, and for the path efficiency at D4, due to large standard deviations. Values are shown as mean ± SD. #significantly different from day 1 at P<0.05.

**Fig 6:**
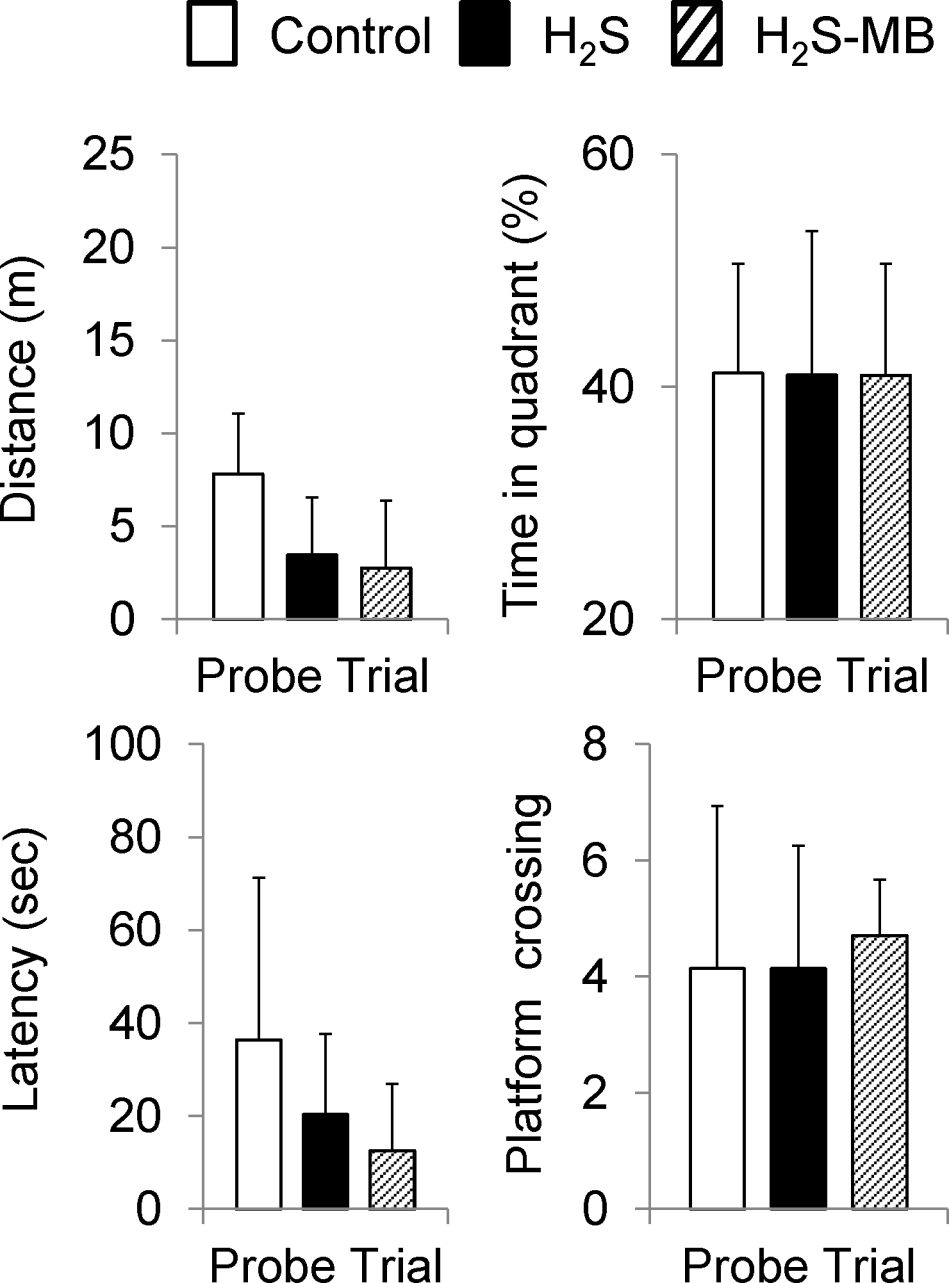
Probe trials. Average latency, distance, time spent in the platform quadrant, and number platform crossing are displayed during the probe trial. No significant difference was observed between the 3 groups. Values are shown as mean ± SD.

Daily averaged swimming distance, latency and path efficiency to find the platform are shown on Fig 5. On the 1^st^ day of training, the control group of 21 rats spent an average of 61.8 ± 42.4 sec in the pool to find the platform; they did not find it within 120 sec in 21% of the tests. The total swimming distance was 13.8 ± 9.3 m and path efficiency was 0.14 ± 0.17. The latency decreased very rapidly at D2 and D3 reaching 23.1 ± 25.3 sec (P<0.05) at D4. The total distance also decreased over time averaging 5.0 ± 5.3 m (P<0.05) at D4, while path efficiency reached 0.44 ± 0.33 (P<0.05) as illustrated on Fig 5. Probe test data are shown on Fig 6.

**Fig 7:**
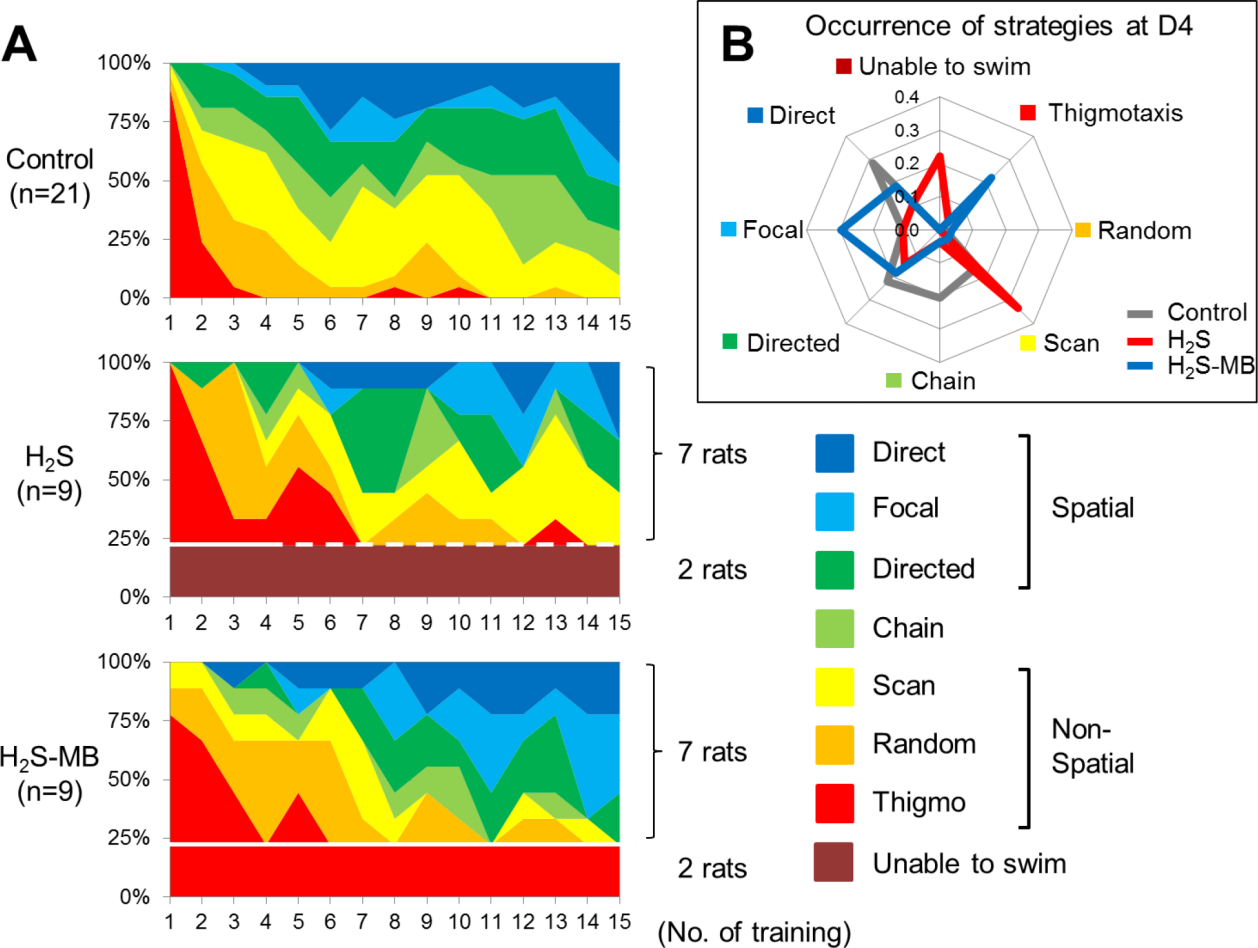
Frequency distribution of the strategies used during the MWM test. **Panel A:** Coding color showing the search strategy used by the Control group (top), H_2_S group (middle), and H_2_S-MB group (bottom) (based on coding proposed by Garthe et al. [54]). Of note, is that the two rats in the H_2_S group unable to swim (#16 and 38, see Table 1) are identified with the dotted line after D2, as they were euthanized at 48 hours. Also in the H_2_S-MB group, 2 rats, although able to swim were only showing a thigmotaxis pattern during the 4 days of training are identified. **Panel B:** Radar chart displaying the relative frequency of the various strategies used at D4 by the 3 groups. In the H_2_S group, the animals used a significantly less effective pattern consisting in higher occurrence of scanning strategy to find the platform when compared to the treated group.

In the H_2_S group, two rats with an obvious motor or visual deficit were unable to swim (Fig 7). All the 7 other rats could swim and their latency to reach platform was not different from the control group averaging 53.7 ± 41.5 sec on the first day (Fig 5). The total swimming distance was 13.4 ± 9.6 m and path efficiency averaged 0.15 ± 0.15, which were similar to the control group, including during the probe trial (Figs 5 and 6). The latency and total distance decreased day by day but this difference was only significant for the distance at D3 and D4, and the path efficiency at D4, due to the large standard deviations.

In the H_2_S-MB group, two out of 9 rats that survived were unable to find the platform, even during the last day of training, although they could swim and had an apparent normal behavior in the open field. The 7 other rats did not show any difference when compared to the control group or the H_2_S group, in terms of the total swimming distance and path efficiency (Figs 5 and 6).

##### Strategy used to find the platform

In the control rats, thigmotaxis disappears almost completely after the first day and the frequency of spatial memory-dependent strategies (direct, focal, and directed search) increased during the period of training (Fig 7). Spatial strategies were used in 17% of trials at D1 versus 66% of trials at D4 (P<0.01). The rats showed a clear preference for direct swimming (29% of trials) as the most efficient strategy of search at 4^th^ day.

**Fig 8:**
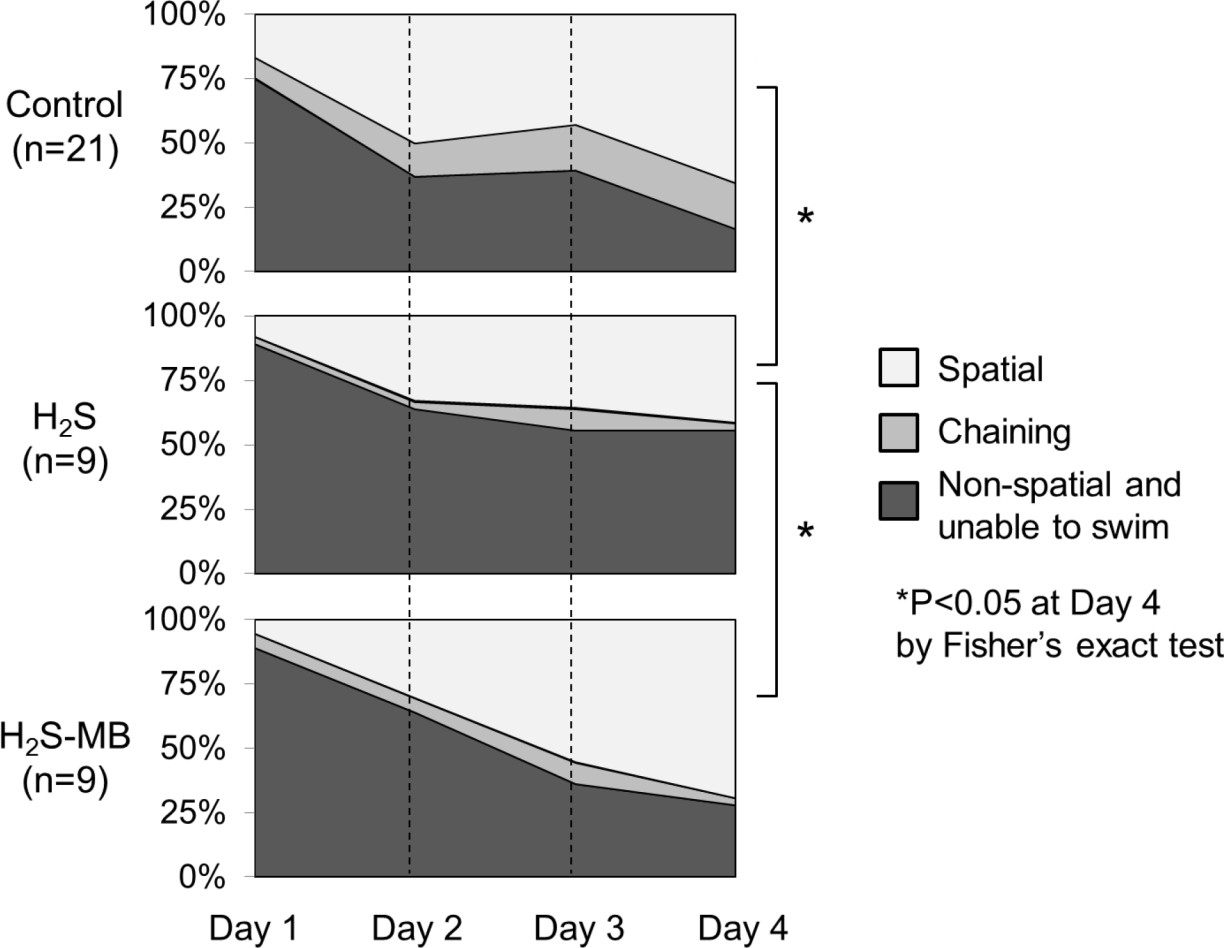
Daily evolution of the spatial dependent and non-dependent strategies used during the MWM test. Data of the Control group (top), H_2_S group (middle), and H_2_S-MB group (bottom) are displayed. All groups showed a progression towards more spatial patterns during the training phase, but spatial strategy was used much less often in the H_2_S group.

In the H_2_S groups, the rats showed typical thigmotaxis swimming pattern on the 1^st^ day of training, like the control group. Spatial strategies were used in 11% of trial at D1 and in 54% of trials at D4 of the rats able to swim, when the analysis included all the rats that received H_2_S, comprising the rats unable to swim as one of the pattern of responses (zero score), only 42% of the rats exposed to H_2_S and surviving the coma were actually able to find a platform using a spatial strategy (Figs 7 and 8) at D4 (P<0.01, by Fisher’s exact test). In addition, the H_2_S exposed rats showed a clear preference for scanning (33% of trials) at D4 in contrast to 16% in the control group (P<0.01, by Fisher’s exact test). Finally, in major contrast to the control group, direct search was used in only 11% of trials in the H_2_S group.

**Fig 9:**
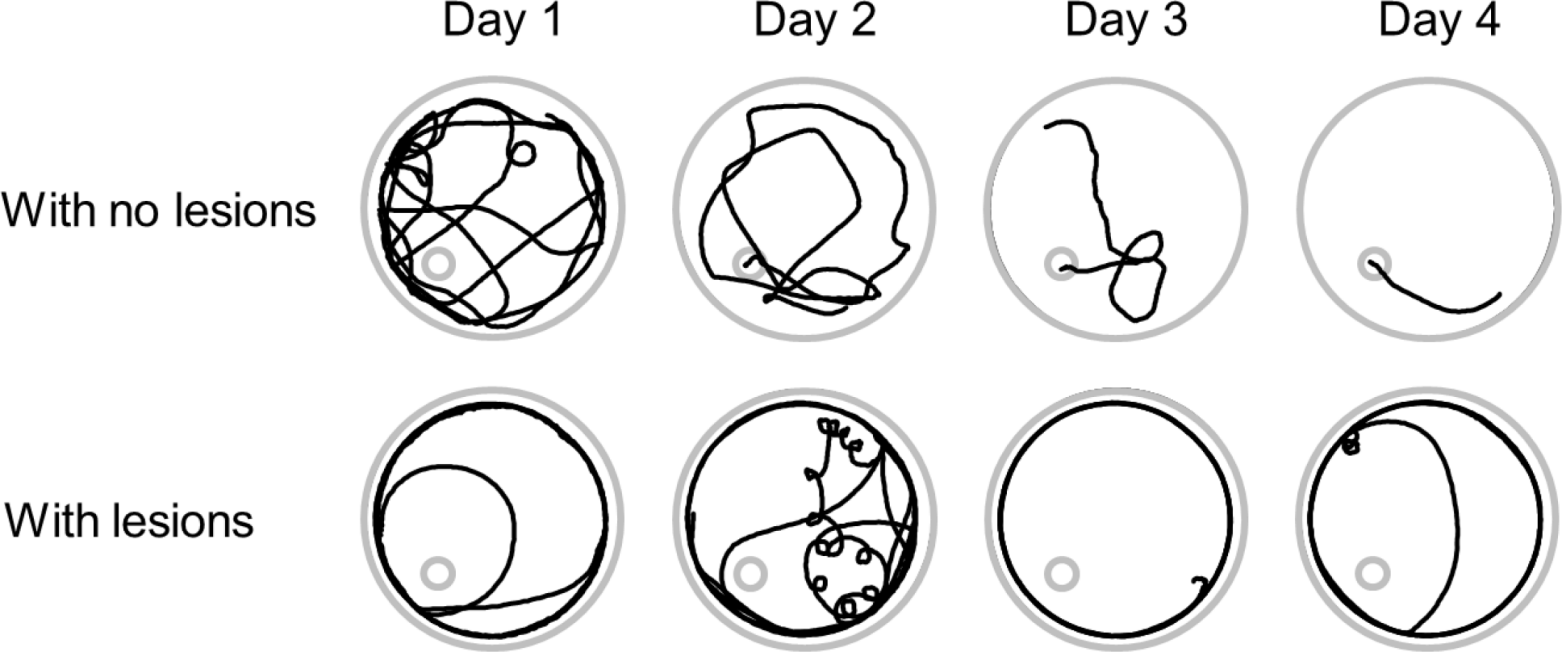
Examples of swimming strategies in 2 intoxicated rats treat with MB. The recordings in the upper panels where obtained from a rat with no brain lesions while those in the lower panels were obtained in the rat #92, (Table 1) that was later found to have neuronal cortical necrosis. The latter rat was unable to find the platform even though its behavior was normal during open field test.

In the H_2_S-MB group, two rats that could not find the platform at D1 and D2 were still unable to find the platform and to engage into any search strategy at the end of the 4^th^ day. These 2 rats also displayed on a few occasions irregular spiral (repetitive looping) swimming patterns (Fig 9). The 7 remaining rats that could find the platform used spatial strategies with a significantly higher occurrence than in the H_2_S group; even after including the animals unable to find the platform, these strategies were present in 69% of trials at D4 (P<0.05 vs H_2_S group). When all the surviving animals were included, the rats presented 19% of direct, 30% of focal, and 19% of directed swimming strategy as spatial memory-dependent strategies during the 4^th^ day, and only 4% of scanning swimming strategy (Fig 7), which was the most frequently used in the H_2_S group (P<0.0001).

In summary, the H_2_S-MB group utilized spatial search oriented patterns as much as control animals, in contrast to a significant decrease in spatial search patterns observed in the H_2_S group. In addition, scanning pattern in both the control group and the H_2_S-MB group were never used as the preferred strategy in contrast to the H_2_S group. This difference remained significant when all rats were considered, including those unable to swim or unable to find the platform (Figs 7 and 8). A final observational note, all surviving rats in the H_2_S-MB group were able to swim, while 2 out of 9 could not in the H_2_S group.

#### g- Brain histopathology

All brains from the rats exposed to H_2_S and MB were examined. The brains of 2 control rats evaluated histologically were used as reference.

**Fig 10:**
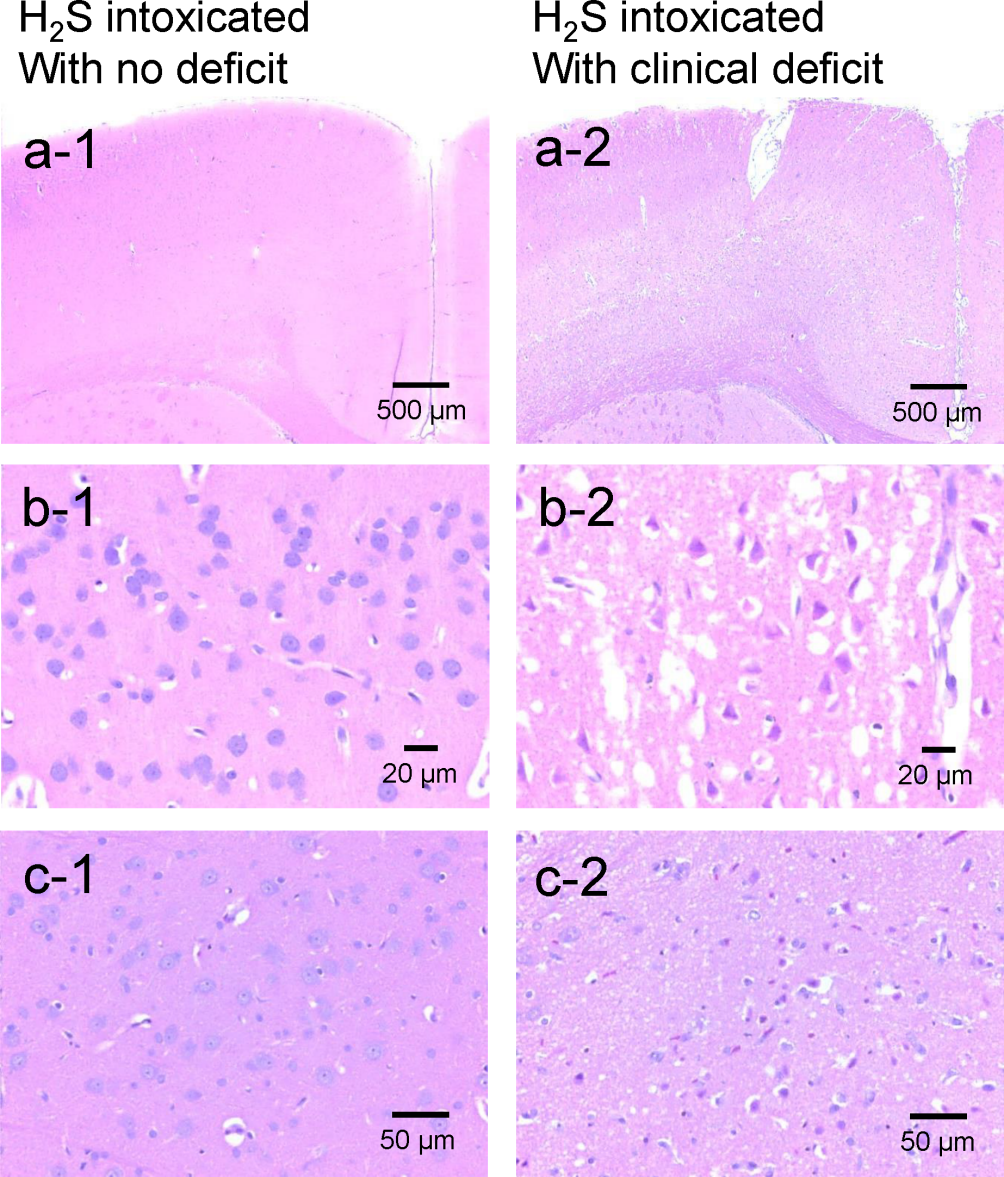
Brain histopathology. Sections of frontal cortex (panels a and b) and thalamus (c) from one rat that presented a coma but with no neurological deficit (a1, b1, c1) and from rat (#16, Table 1) that was unable to swim after H_2_S exposure (a2, b2, c2). In contrast to rat with no symptom, the brain of rat #16 showed diffuse and extended neuronal necrosis and neuropil edema affecting the outer frontal cortex (motor agranular cortex) and the cingulate gyrus (anterior limbic area). Neurons are hypereosinophilic with karyolytic or pyknotic nuclei and peri-nuclear edema, Bregma 0.0. 400x. Panel c1 shows normal thalamus at same level and magnification in the intoxicated rat with no deficit. Panel c2: Extensive neuronal necrosis in the lateral posterior nucleus of the thalamus. Bregma -4.8. 400x.

**Fig 11:**
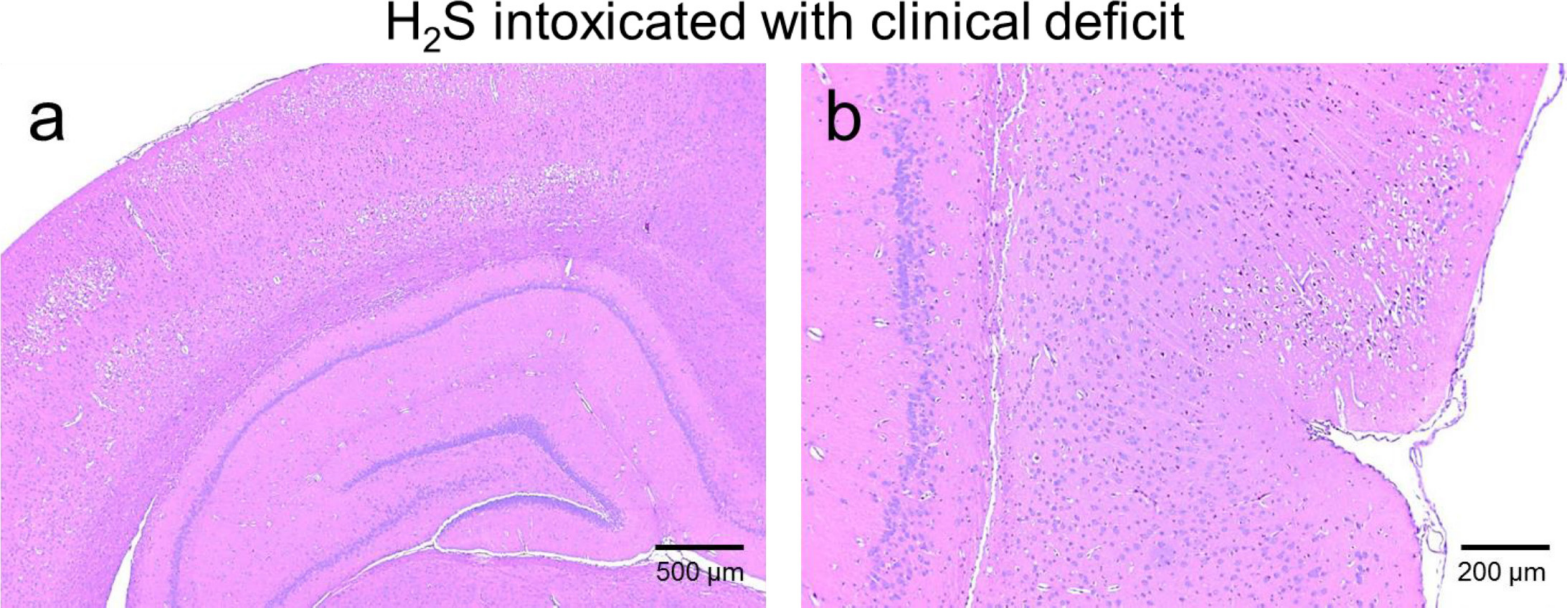
Histopathology of the hippocampus and piriform cortex in the rat # 38 that displayed severe neuronal necrosis in the frontal, temporal, parietal and occipital cerebral cortices. The same pattern was found in all the rats presenting cortical lesions. a) Note the diffuse acute neuronal necrosis and neuropil edema affecting the outer retrosplenial and occipital cortex, while the hippocampus was completely unaffected. Bregma -4.5. 40x; b) Note the sharp demarcation at the rhinal fissure between the necrotic temporal cortex (top) and the unaffected piriform cortex (bottom). Bregma -4.5. 100x.

**Fig 12:**
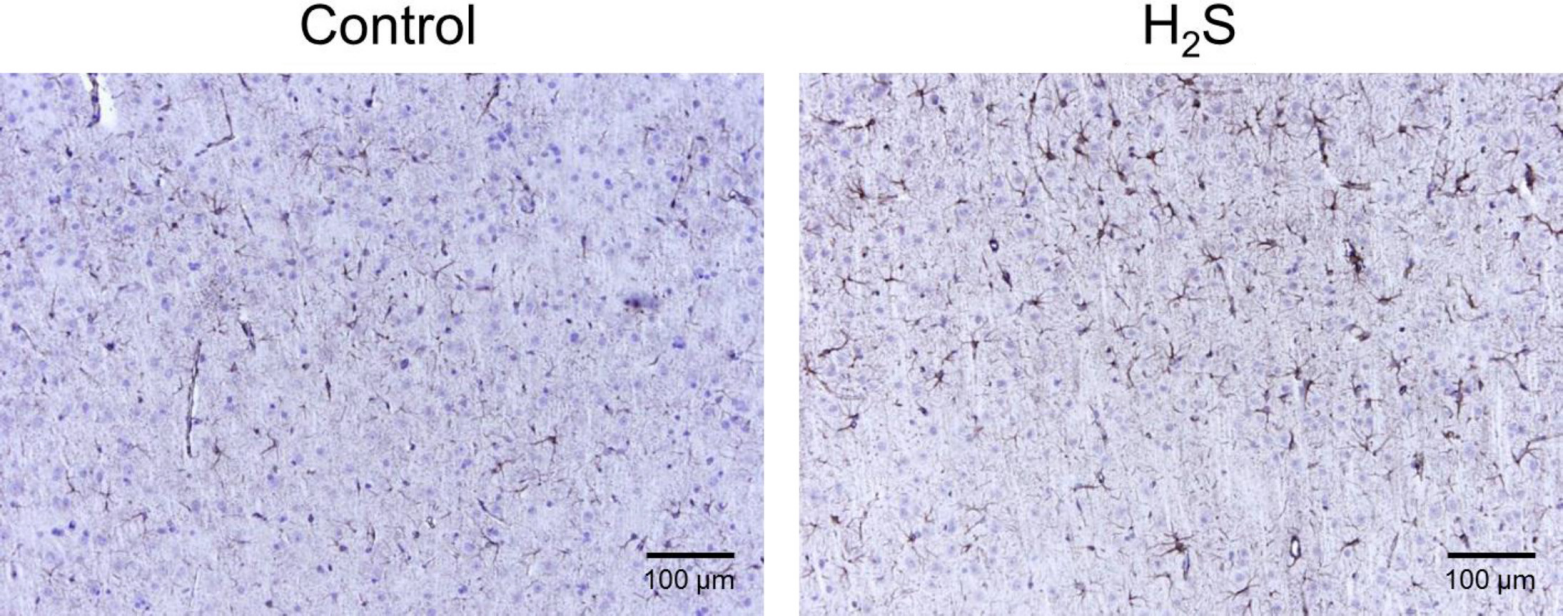
GFAP staining by DAB chromagen with hematoxylin counterstain in one rat exposed to saline (control) and one rat intoxicated with H_2_S with normal recovery. There was no difference in astrocyte density or morphology between the 2 animals.

For the H_2_S group, in the 7 rats that presented no clinical deficit, no brain lesions (necrotic or apoptotic neurons) were found (Fig 10A-1, 10B-1 and 10C-1). In contrast, the two rats that had to be euthanized at 48 hours following the NaHS injection due to their inability to eat and persistent motor deficit, presented extensive necrotic lesions consisting in severe bilaterally symmetric, multifocal neuronal necrosis of the superficial and middle laminae of the frontal, parietal, temporal and occipital lobes of the cerebral cortex and cingulate gyrus, with sparing of the deepest laminae (Fig 10A-2, 10B-2 and 10C-2). Neuronal necrosis was also present but more variable in the caudate putamen, amygdala and thalamus (Table 1). The piriform cortex, cerebellar Purkinje cell layer and hippocampal (CA1-3 and dentate gyrus) neuronal populations were completely unaffected. The white matter tracts in the cortex were also unaffected. Examples are shown in Figs 10 and 11, while Table 1 summarizes the location of the various lesions. The brains from all four rats that received H_2_S, but that displayed no necrotic lesions were negative for cleaved caspase-3. The number of GFAP positive astrocytes and Iba1 positive microglia was not different from the control animals, (Fig 12).

**Table 1:** Summary of location of the various brain lesions.

	H_2_S		H_2_S-MB		
Rat ID	16	38	89	92	98
Cerebral cortical grey matter:	Massive diffuse symmetric necrosis of superficial and middle laminae	Massive multifocal necrosis of superficial and middle laminae	Massive symmetric, diffuse necrosis of superficial and middle laminae	Massive *asymmetric*, diffuse necrosis of superficial and middle laminae	Massive *asymmetric*, diffuse necrosis of superficial and middle laminae
cingulate gyrus					
frontal					
parietal					
temporal					
occipital					
piriform	Unaffected (sharp demarcation with adjacent severely necrotic parietal and temporal cortex)		Unaffected	Rostral portions were affected	Unaffected
Olfactory bulb	Unaffected		Bilaterally symmetric necrosis within the dorsal portions	Asymmetric necrosis	Unaffected
Cortical white matter	Mild edema and acute hemorrhage	Unaffected	Unaffected		
Anterior commissure and corpus callosum	Unaffected		Unaffected		
Caudate putamen	Multifocal necrosis affecting approximately 5% of neurons	Multifocal necrosis affecting approximately 2% of neurons	Unaffected	Rare apoptotic neurons	Unaffected
Hippocampus (Cornu ammonis and dentate gyrus)	Unaffected		Unaffected		
Thalamus	Focal necrosis in the lateral posterior nucleus	Unaffected	Focal neuronal necrosis in the reticular nucleus	Extensive and asymmetric necrosis	Neuropil vacuolation and gliosis in ventroposterolateral nucleus
Amygdala	Focal necrosis in the cortical nucleus	Multifocal necrosis affecting approximately 2% of neurons	Unaffected		
Cerebellum including Purkinje cell layer	Unaffected		Unaffected		

**Fig 13:**
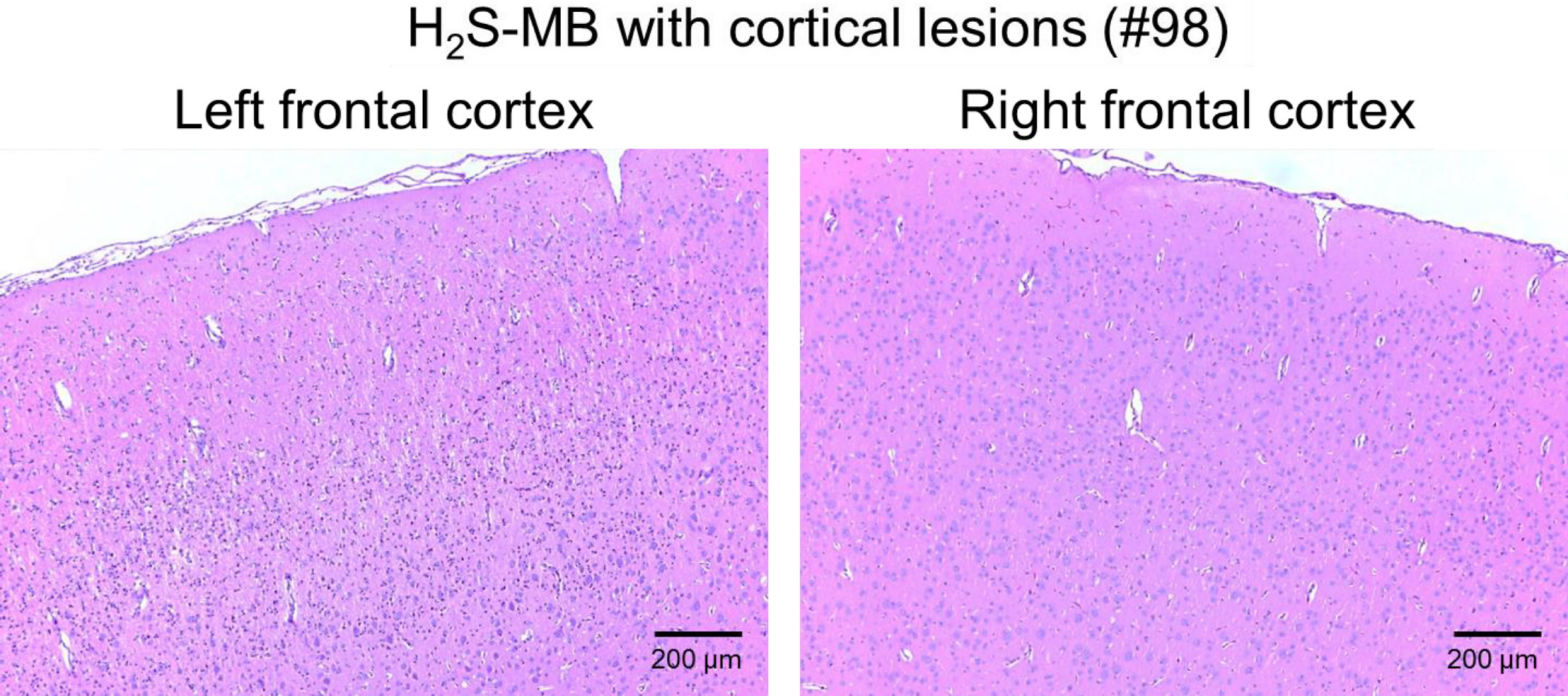
Histology of the frontal cerebral cortex in one rat of the H_2_S-MB group (#98, see Table 1) that could swim and find the platform. Marked asymmetry of neuronal necrosis in the left hemisphere was found, while the right hemisphere was nearly normal (Bregma 0.0. 40x). All other lesions in this rat (Table 1) were ipsilateral.

In H_2_S-MB groups, the 2 rats that were unable to find the platform presented the same types of lesions as the 2 rats that had to be euthanized in the H_2_S group. To our surprise, one of the 7 rats able to find the platform had significant brain lesions (Fig 13), while the brains of the other 6 animals were normal. This rat displayed notably asymmetrical neuronal necrosis of the superficial and middle laminae of the cerebral cortex (the frontal, occipital, parietal and temporal lobes) and the cingulate gyrus. The piriform cortex, corpus callosum, caudate putamen and hippocampus were unaffected.

**Fig 14:**
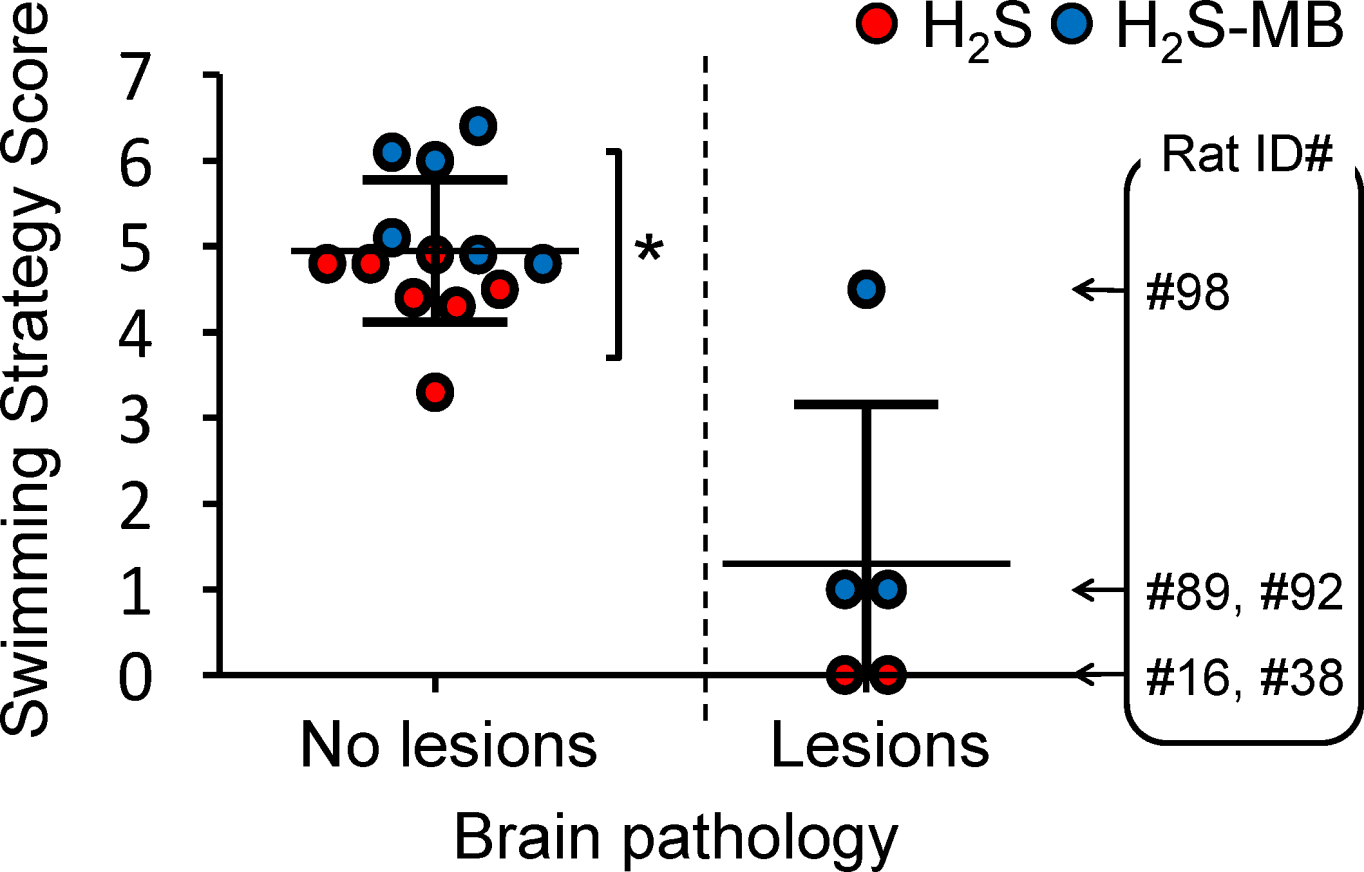
Average swimming strategy score in all the intoxicated rats with and without brain lesions following H_2_S induced coma in non-treated animals (red symbols) and in animals receiving MB (blue symbols). The swimming strategy was scored according to the swimming pattern described in the Fig 1 (the rats unable to swim were scored as zero), the score was averaged over the last 2 days of training. The score of the rats reaching the platform was significantly higher in the treated group than in the non-treated animals (*P<0.01, see text for further details). The ID numbers of the rats presenting with brain lesions (corresponding to the ID numbers in Table 1) are shown. In all rats but one, lesions were highly predictable from the clinical picture.

Swimming strategies were scored according to the swimming pattern presented in Fig 1 (from 0 unable to swim to 7 direct swimming). The scores were averaged over the trials performed in the last 2 days. The 13 rats with no brain lesions scored 4.9 ± 0.8, while the 5 rats with severe brain lesions displayed an average score of 1.3 ± 1.9 (Fig 14). Finally, the score of the rats reaching the platform was significantly higher in the treated group than in the non-treated animals (5.6 ± 0.7 versus 4.4 ± 0.5, P<0.01).

#### h- Additional observations

**Fig 15:**
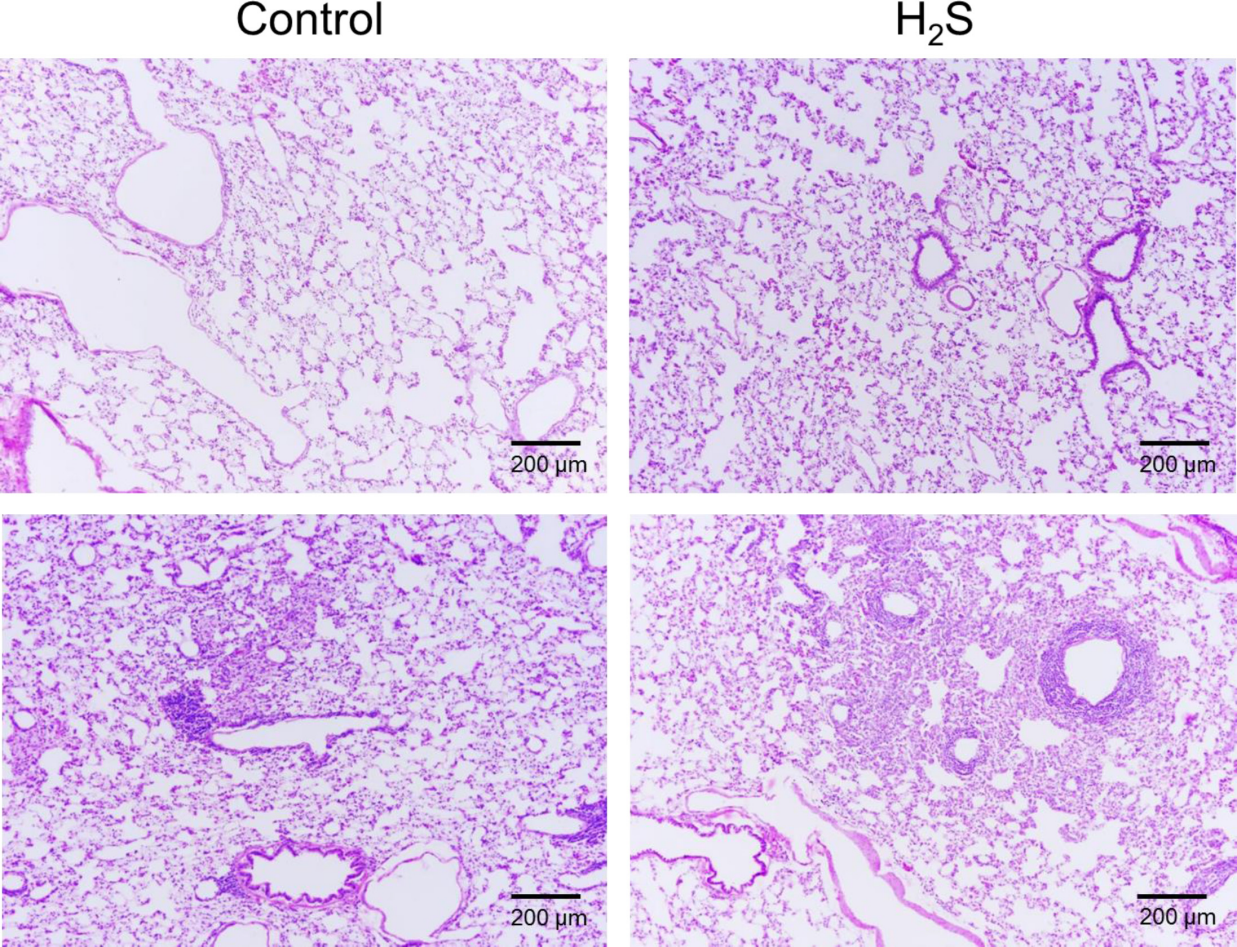
Example of the histopathology of the lung in a control rat and a rat exposed to H_2_S. Hematoxylin and eosin stained sections of perfused lungs from control and H_2_S-exposed rats, 100X magnification. Lungs of both rats are normal (top panels) with no evidence of lesions that would be expected to occur during inhaled sulfide exposure. As illustrated on the lower panels, both unexposed controls and H_2_S exposed rats displayed mild expansion of the peribronchial, peribronchiolar and perivascular interstitium by low to moderate numbers of lymphocytes and macrophages with fewer plasma cells consistent with chronic bronchus associated lymphoid tissue hyperplasia (BALT, see text for additional comments).

None of the rats exposed to H_2_S displayed any pulmonary lesions, including in the airways, compatible with sulfide toxicity, such as necrosis of the respiratory epithelium [56] or evidence for pulmonary edema [57] as shown in Fig 15. Of note is that most of the control and sulfide exposed rats displayed features consistent with chronic bronchus associated lymphoid tissue (BALT) hyperplasia which was regarded as nonspecific findings, common in rats with pulmonary colonization by various bacterial agents [58].

## Discussion

This study showed that, in a rat model, H_2_S-induced coma resulted in a global mortality rate of 60% by cardiac arrest. The majority of the surviving animals recovered without any obvious clinical deficit within the first 48 hours and were able to swim and locate the hidden platform in Morris Water Maze as fast as the control rats. These animals did not display evidence for neuronal necrosis or memory deficit. However, they use a different searching pattern, indicating subtle but measurable deficits in their ability to use more direct spatial strategies to perform their task. Twenty-two per cent of the surviving animals were unable to swim due to motor or visual deficit and were found to have massive and diffuse cortical necrosis. Interestingly, the necrosis presented with a topology different from that typically observed after severe anoxia or ischemia. MB injected within one minute into the phase of coma—typically when gasping was produced- dramatically increased the number of animals that survived and were able to perform the MWM tasks. Twenty-two percent of the surviving animals, although capable of swimming and with a normal behavior in the open field were never able to find the platform. They presented lesions similar to those found in the rats unable to swim in the H_2_S groups. In addition, one animal, which behaved just like the control animals in the open field and was able to find the platform, presented neuronal necrosis, which were clearly unilateral. All the other MB treated animals had no brain lesions.

### Rat model of H_2_S-induced coma

H_2_S is one of the most toxic gases with potentially dreadful effects on neurons due to their inability to oxidize H_2_S [59, 60]. Long term effects on cognitive function and memory in patients surviving H_2_S induced coma have been described in case reports [6, 7, 61, 62], but their mechanisms, i.e. direct toxicity of H_2_S by blocking the cytochrome C oxidase activity or potentiation of sulfide toxicity by anoxia or ischemia produced by the cardiorespiratory failure remain debated [1, 10]. Our goal was to use this rat model in order to both evaluate of the immediate outcome and the long-term effects on spatial memory and learning processes in the surviving rats. In addition, our model allowed us to evaluate the beneficial effects of MB as a potential treatment of H_2_S poisoning. However using systemic rather than inhaled administration creates specific conditions that may differ from real life exposure wherein the airways and the lung parenchyma are exposed to higher concentration of sulfide [57]. Complications in the form of lesions of the upper airways [56] and pulmonary edema [57] are therefore less likely to develop and were not produced in our model (Fig 15).

We used an approach that was different from the previous rat models of sulfide poisoning induced coma [10, 42] by targeting our focus of investigation to the outcome of the coma, rather than that of sulfide poisoning per se. Due to the high lethality of H_2_S and the thin margin between innocuous levels [63], those producing a reversible coma and those producing a coma leading to a rapid cardiorespiratory arrest, we used an approach wherein the coma was produced in about 30% of cases after one injection [28]. Due to the almost immediate disappearance of sulfide after the end of exposure [27, 63–65], a second and third injection could be performed with no clear cumulative effects, because of the rapid oxidation of sulfide in the blood [63, 66] and in the tissues [22, 60, 67]. We found no evidence for a dose-response phenomenon between 1 or 3 IP injections in terms of lethality. In order to produce a coma, H_2_S concentrations in the blood and the brain must reach a “toxic” threshold [65], which depends on the rate of diffusion of the solution of H_2_S/HS^-^ into the blood through the peritoneal membrane which we believe can account for this unpredictable outcome. Yet, the global mortality rate of our model (60%) is comparable to what was found by Warenycia et al. [26] and that produced with a similar model using only 2 IP injection [28]. The mechanism of immediate death was a cardiac arrest, resulting from an acute decrease in cardiac contractility [28] leading to a pulseless electrical activity (PEA) [64, 68]. The mechanism of this rapid and dramatic cardiac depression is not clearly established and may relate to the known inhibitory effects of H_2_S on cytochrome C oxidase leading to a reduction in ATP production and impeding cardiomyocytes contractions. These effects may also be accounted for by H_2_S induced L-type calcium channel blockade [69, 70], a potentiation of the depressive effects of NO on cardiac contractility [71, 72] or the production of free radicals during sulfide exposure [73, 74]. Of note is that in all animals heart stopped when animal were still presenting gasps, suggesting that H_2_S induced apnea was not the mechanism for cardiac arrest.

### Outcome in the Surviving animals

Most rats that did not present a cardiac arrest recovered completely within 20 minutes from their coma and were able to take part of the Morris Water Maze task. This picture is very similar to the clinical description of knockdown wherein subjects lose consciousness but can recover with no apparent deficit after an acute H_2_S exposure [2, 3, 9, 75]. In these rats, the spontaneous motor activity was strictly similar to control rats, although there was a statistically significant higher loss of weight in the animals that had presented a coma (on average 13 ± 11 g, i.e. 2.6 ± 2.2% of the initial body weight). The picture was very different in the rats that did present with motor or sensory deficits along with extensive brain necrosis. The reason for this large spectrum of after-effects is not clear, but it should be kept in mind that although knockdown is typically reversible, severe cognitive, sensory or motor deficit can develop in patients with more severe form of coma, or when ischemia is present due to a transient low blood pressure or cardiac output[8]. The lesions observed in these animals were very impressive and consisted of neuronal necrosis of the superficial and middle laminae of the frontal, parietal, temporal and occipital lobes of the cerebral cortex and cingulate gyrus, with sparing of the deepest laminae. Less severe/extensive necrotic lesions were also found in the caudate putamen, thalamus as well as amygdala. The piriform cortex, cerebellar Purkinje cell layer and hippocampal (CA1-3 and dentate gyrus) neuronal populations were not affected. Since anoxia or hypoxia-induced brain damage is well known to involve deeper layers of cerebral cortex along with hippocampal and neocortical pyramidal cells, striatal neurons, and Purkinje cells [76–78], lesions secondary to H_2_S-poisoning appear to have a different anatomical distribution. H_2_S being transported by the blood could have produced lesions in regions with high metabolism and high perfusion, rather than in the deeper watershed areas where perfusion is compromised, in contrast to the effects of ischemia. The lack of obvious memory deficit in the surviving animals correlates well with the absence of hippocampal lesions in the sickest animals, while the motor deficit and blindness observed in the 2 rats belonging to the H_2_S group, can be accounted for by lesions affecting the thalamus, the motor or visual cortex as well as subcortical nuclei (Table 1).

In humans, who are severely intoxicated with deep coma associated with shock, studies using neuroimaging have shown decreased metabolism of the putamen, basal ganglia and cortical atrophy, including the motor cortex [6, 8, 62]. Similarly, neuronal lesions developing after severe forms of sulfide intoxication in cattle found in a pit containing lethal levels of sulfide [79], appear to share some similarity with those reported in the present study but also presented necrotic lesions closer to those observed following ischemia, i.e. affecting the deeper layers of the cortex, the hippocampus and the Purkinje cells of the cerebellum. These results suggest that in many of these observations, a significant component of post anoxic/ischemic injury is present. It should be kept in mind that H_2_S appears to be able to prevent the increase in HIF-1alpha produced by hypoxia [80], therefore making cells exposed to H_2_S defenseless against some of the effects of ischemia.

### Morris Water Maze

After being introduced to the MWM pool, most animals showed a behavior known as ‘‘thigmotaxis” [81]. This pattern is rapidly replaced by a similarly non-spatial ‘‘random search” or “scanning” strategies, characterized by an absence of directional preference (Fig 1). After only 4 training sessions (D1), the search behavior became restricted to the central region of the pool. Subsequent trials result in rats tending to use a more allocentric/spatial mode of navigation consisting of ‘‘focal search” or ‘‘direct swimming” aiming at the platform, regardless of the starting position. H_2_S-intoxicated rats were able to find the hidden platform within the same time as the control group and did not display evidence for alterations in spatial memory and the learning processes [54, 82, 83]. This observation is consistent with the absence of lesion of the hippocampus in the 2 sickest animals. However, the intoxicated animals, that did not received MB, used a different pattern of search; less spatial strategies were adopted. It is not clear what would be the clinical equivalent of such a pattern in humans. Visual deficit, reported in patients after H_2_S poisoning [7], may well manifest by a non-spatial strategy without altering the time to reach the platform, as even blind rats do find a hidden platform with a latency that appear to be similar to normal rats [84, 85]. It should be kept in mind that vision in albino rats is already altered and even moderate visual deficit may well result in a scanning strategy used more often. In addition, lesions affecting the putamen can alter MWM performance [86, 87], lesions also observed in humans after sulfide intoxication [8].

### Effects of MB

MB reduces the immediate mortality following acute exposure to sulfide by preventing the rapid development of PEA [28]. The resulting higher proportion of surviving animals in the MB treated group than in non-treated animals creates a bias preventing the proper analysis of the neurological outcomes, since for obvious reasons, only the surviving animals were studied in both groups. Yet, a significantly higher proportion of spatial strategy search in the treated group was found. In addition, the 2 animals that displayed cortical and subcortical necrotic lesions in the MB groups, akin to the lesions found in the 2 non-treated animals (#16 and #38 see Table 1) were able to swim. Even more intriguing is that one of the MB treated animals (#98 in Table 1) had a normal behavior in the MWM and was found to have asymmetric lesions, a topography difficult to explain.

MB has a central aromatic thiazine ring system, which confers to this agent a high lipophilicity and the ability to concentrate in the heart and central nervous system after systemic injection. Its pharmacokinetics, both in the blood and tissues, has been established in various species including in humans [88, 89]; MB has been shown to have a relatively long half-life in the blood and to diffuse extremely rapidly into most tissues including the brain [89], where it accumulates. Inside the cells, MB concentrates in the mitochondria where it can interact with the respiratory complexes. Part of MB is reduced into leuco-methylene blue forming with MB a reversible oxidation-reduction system or electron donor-acceptor couple [90, 91] (see also [33, 36]).

MB appears to support the transfer of protons through the mitochondrial membrane against a concentration gradient, essential for the production of adenosine triphosphate (ATP), and can bypass the normal electron flow, when mitochondrial respiration is impeded [30–33]. This effect clearly antagonizes the mechanism of hydrogen sulfide toxicity [59, 92–94] and has already been suggested to reduce post-anoxic brain injury produced by a cardiac arrest [90, 95–98]. As mentioned in the introduction, MB has been also shown to exert a remarkable protection against the toxic effects of sodium azide (SA) [40], which is a poison of the mitochondrial activity.

To which extend H_2_S lethality is the result of a deficit in ATP is not that clear, indeed as discussed above, H_2_S could exert its toxicity on the heart through non-ATP related mechanisms. These mechanisms include the acute blocking of calcium channels [69, 70], the potentiating the depressive effects of NO on the heart [71, 72] as well as via the production of free radicals [73, 74]. MB at low doses exerts a potent anti-nitric oxide (NO) effect [37, 38], as it inhibits guanylyl cyclase (GC), leading to a decrease in cyclic guanosine monophosphate (cGMP) [38], i.e. the second messenger used by NO to transduce its cellular effects [99, 100]. This anti-NO effect has been demonstrated in many animal models and is the basis for treating refractory shock in humans, including post-operative vasoplegia or anaphylactic shock [101–107] and to a much lesser extent septic shock [108–111], wherein intracellular GC is activated, presumably under the influence of NO. These effects can be understood in the light of the recent results demonstrating a strong positive interaction between NO and H_2_S-induced vasoplegia [112–114].

The potent anti-oxidant properties of MB at low doses may also have contributed to the protection of selective regions of the brain [33]. These anti-oxidant effects of MB [33–36] have been proposed to account for the preservation and restoration of cognitive deficit in various models of brain dysfunction-induced amnesia [33].

MB would represent an approach to treat H_2_S poisoning that is very different from the traditional paradigm relying on the use of metallo-compounds containing Fe^3+^ [20, 22] or Co^3+^ [19, 22, 115] aimed at combining and “neutralizing” free/soluble H_2_S. The use of the current proposed antidotes is limited by the very rapid disappearance of soluble H_2_S following hydrogen sulfide poisoning [65].

The fundamental question remains on the exact nature of the mechanism of “protection” of the cardiac function and the central nervous system, which could account for the immediate reduction in mortality in the MB treated group and the apparent better outcome that we observed during the MWM testing.

In conclusion, the majority of rats surviving H_2_S induced coma showed a transient reduction in weight, but no reduction in their spontaneous activity and no evidence for alteration in spatial memory. A difference in search strategy towards less direct non-spatial patterns was however present. In few animals, a motor or sensory deficit associated with major necrotic lesions in the cortical structures, basal ganglia and the thalamus were present. MB administration during the phase of gasping dramatically increased the survival of animals with no clinical deficit, and it increased the use of spatial search strategy during MWM testing trials. Yet, about the same percentage of surviving animals displayed brain necrotic lesions, which in one case was unilateral, but presented less severe clinical manifestations. The location and distribution of necrotic lesions differed from those expected to be found in post-ischemic brain injury. This model produces a range of manifestations and outcomes similar to those observed in humans and can be used to test the benefit of antidotes or symptomatic treatments of sulfide poisoning that would be most relevant to the clinical setting.
